# Unraveling the interplay between root exudates, microbiota, and rhizosheath formation in pearl millet

**DOI:** 10.1186/s40168-023-01727-3

**Published:** 2024-01-03

**Authors:** Abdelrahman Alahmad, Mourad Harir, Sylvain Fochesato, Joris Tulumello, Alesia Walker, Mohamed Barakat, Papa Mamadou Sitor Ndour, Philippe Schmitt-Kopplin, Laurent Cournac, Laurent Laplaze, Thierry Heulin, Wafa Achouak

**Affiliations:** 1 CEA, CNRS, BIAM, Lab Microbial Ecology of the Rhizosphere (LEMiRE), Aix Marseille Univ, 13108 Saint-Paul-Lez-Durance, France; 2 UniLaSalle, SFR NORVEGE FED 4277, AGHYLE Rouen UP 2018.C101, 3 Rue du Tronquet, 76130 Mont-Saint- Aignan, France; 3 Research Unit Analytical BioGeoChemistry, Helmholtz Munich, Ingolstaedter Landstrasse 1, 85764 Neuherberg, Germany; 4 Chair Analytl Food Chem, Technical University of Munich, 85354 Freising, Weihenstephan Germany; 5 CIRAD, INRAE, Eco&Sols, Université de Montpellier, Institut Agro, IRD FR, Montpellier, France; 6 UCEIV-ULCO, 50 Rue Ferdinand Buisson, 62228 Calais, France; 7 LMI IESOL, Centre de Recherche, ISRA-IRD de Bel Air, Dakar, Senegal; 8 UMR DIADE, Université de Montpellier, IRD, CIRAD, Montpellier, France; 9 LMI LAPSE, Centre de Recherche, ISRA-IRD de Bel Air, Dakar, Senegal

**Keywords:** Pearl millet, Exudates, Soil aggregation, Microbiota, Metabonomics

## Abstract

**Background:**

The rhizosheath, a cohesive soil layer firmly adhering to plant roots, plays a vital role in facilitating water and mineral uptake. In pearl millet, rhizosheath formation is genetically controlled and influenced by root exudates. Here, we investigated the impact of root exudates on the microbiota composition, interactions, and assembly processes, and rhizosheath structure in pearl millet using four distinct lines with contrasting soil aggregation abilities.

**Results:**

Utilizing 16S rRNA gene and ITS metabarcoding for microbiota profiling, coupled with FTICR-MS metabonomic analysis of metabolite composition in distinct plant compartments and root exudates, we revealed substantial disparities in microbial diversity and interaction networks. The ß-NTI analysis highlighted bacterial rhizosphere turnover driven primarily by deterministic processes, showcasing prevalent homogeneous selection in root tissue (RT) and root-adhering soil (RAS). Conversely, fungal communities were more influenced by stochastic processes. In bulk soil assembly, a combination of deterministic and stochastic mechanisms shapes composition, with deterministic factors exerting a more pronounced role. Metabolic profiles across shoots, RT, and RAS in different pearl millet lines mirrored their soil aggregation levels, emphasizing the impact of inherent plant traits on microbiota composition and unique metabolic profiles in RT and exudates. Notably, exclusive presence of antimicrobial compounds, including DIMBOA and H-DIMBOA, emerged in root exudates and RT of low aggregation lines.

**Conclusions:**

This research underscores the pivotal influence of root exudates in shaping the root-associated microbiota composition across pearl millet lines, entwined with their soil aggregation capacities. These findings underscore the interconnectedness of root exudates and microbiota, which jointly shape rhizosheath structure, deepening insights into soil–plant-microbe interactions and ecological processes shaping rhizosphere microbial communities. Deciphering plant–microbe interactions and their contribution to soil aggregation and microbiota dynamics holds promise for the advancement of sustainable agricultural strategies.

Video Abstract

**Supplementary Information:**

The online version contains supplementary material available at 10.1186/s40168-023-01727-3.

## Introduction

The pressing challenges of rapid population growth and escalating food demands have driven the need for increased agricultural production [1]. However, these efforts are significantly complicated by the complex issues brought on by climate change, including drought, soil degradation, salinity, and pollution. To address these multifaceted challenges effectively, it is essential to identify specific root traits that enhance resource acquisition by plants. Enhanced root systems are instrumental in enabling crops to optimize the utilization of soil resources, offering a pathway to increased productivity and favorable environmental outcomes. Therefore, the recognition and understanding of these particular root traits that facilitate efficient soil resource capture is of utmost importance [2]. Furthermore, within this context, the rhizosheath, the soil layer tightly adhering to the roots, assumes a critical role in assisting plants in their resilience against drought conditions, particularly when the soil experiences moderate dryness [3]. For instance, features such as root branching, the formation of root hairs, and partnerships with arbuscular mycorrhizal fungi have all been connected to some degree with the establishment of a rhizosheath [3, 4]. Furthermore, the composition of root exudates and the exopolysaccharides released by microorganisms associated with the roots exert a significant influence on the stability of the soil aggregates surrounding the root system, as well as on the plant’s water and nutrient uptake [5].

Pearl millet (*Pennisetum glaucum*) is grown in regions with limited agronomic potentials, characterized by low rainfall (100–500 nm) and soils poor in organic carbon, where other crops tend to fail. However, the genetic potential of pearl millet, which can be utilized to enhance its tolerance to abiotic stresses such as water stress and improve yield, has yet to be fully exploited [6]. Encouraging prospects exist for leveraging the available sequenced genome [7] and the identification of yield-associated quantitative trait loci (QTLs) [8, 9] offering promising avenues for further improvement [10]. Pearl millet, despite its immense potential for climate change adaptation in African and Indian agriculture, is often considered an orphan crop with a smaller scientific community compared to other cereals [11–14]. However, deeper insights into soil–plant-microbiota interactions in the root environment can unlock new avenues for sustainable improvement of pearl millet production [15, 16]. The formation of the rhizosheath in pearl millet is genetically controlled and primarily regulated by root exudates [17, 18].

The objective of this study was to establish correlations between the diversity of root-associated microbiota in pearl millet lines, their soil aggregation capacities, and the composition of root exudates under in situ conditions. To achieve this, we employed metabarcoding and metabonomics approaches to identify the microbial diversity on the roots and in the rhizosphere, as well as the nature of root exudates, in four inbred lines of pearl millet grown in natural soil.

This study builds upon previous advancements in pearl millet genomics and presents a valuable opportunity for further improvement and innovation in crop breeding programs.

## Results

### Diversity of the pearl millet root-associated microbiota

**Fig. 1:**
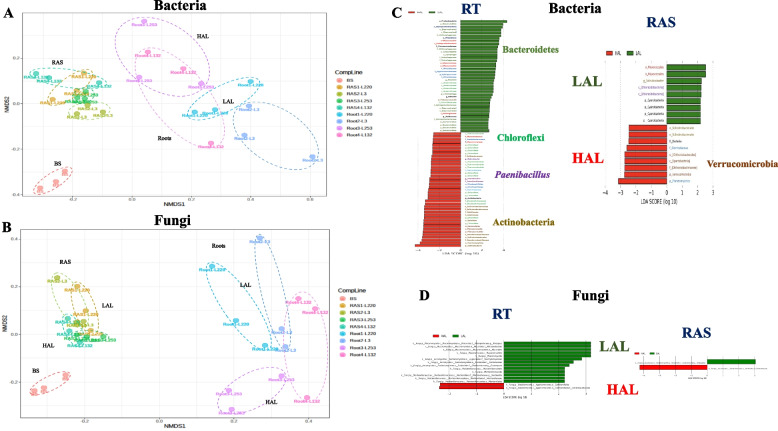
Microbial community beta-diversity and differential taxonomic biomarkers in pearl millet rhizosphere and bulk soil (BS). NMDS plots illustrating the beta-diversity of (**A**) bacterial communities, and (**B**) fungal communities, in the different compartments (root tissues (RT) and root-adhering soil (RAS)) in the four-pearl millet “PM” lines (L220, L3, L253, and L132) along with bulk soil “BS.” The dots correspond to individual samples, where red dots indicate BS samples, green dots represent RAS samples, and blue dots represent root samples. PERMANOVA with a *p*-value < 0.001. **C** Bar chart depicting the results of LEfSe analysis conducted on the roots and RAS bacterial communities, and **D** of fungal communities, of low-aggregation line (LAL, including L220, L3) and high-aggregation line (HAL, including L253, and L132) pearl millet. The chart displays log-transformed LDA scores of bacterial taxa identified by LEfSe analysis, with a threshold of 2.0 for the log-transformed LDA score

The composition of active bacteria and fungi of the root tissues (RT) and root-adhering soil (RAS) fractions of 4 pearl millet (PM) lines contrasting for their ability to aggregate the root-adhering soil (rhizosheath) were examined at phylum level (Fig. S1A–B). According to the ranking of the four PM lines based on the RAS/RT mass ratio, the four PM lines were classified as, low-aggregation lines (LAL-L220 and LAL-L3, RAS/RT = 7.8 and 12.9 g/g, respectively) and high-aggregation lines (HAL-L253 and HAL-L132, 23.6 and 24.8 g/g, respectively) [17]. We observed a significant difference in both bacterial and fungal community composition between different PM lines across compartments (bulk soil (BS), RAS, RT) (PERMANOVA, *F*-value 5.1548,*R*^2^ 0.69614; *p*-value < 0.05 and *F*-value 3.8382; *R*^2^ 0.63044; *p*-value < 0.05, respectively). Non-metric multidimensional scaling (NMDS) plots illustrate these divergences between compartments (NMDS stress = 0.11205 and NMDS stress = 0.14441, respectively). Specifically, RAS samples exhibited distinct microbial communities compared to RT samples and the BS, for both bacteria and fungi (Fig. 1A and B). The major change in bacterial community composition was detected between bulk soil (BS) and RAS, which was contributed by Actinobacteria (average 43 vs 14%), Proteobacteria (average 24 vs 54%), Chloroflexi (average 19 vs 5%), Acidobacteria (average 5 vs 12%), and Firmicutes (average 2 vs 8%). At phylum level of taxonomic resolution, there was no significant difference in the bacterial composition of RAS fraction between the four PM lines. In contrast, bacterial community in the RT was different between PM lines (Fig. S1A). The LAL-L220 and LAL-L3 were enriched in Bacteroidetes (10–13%) compared to HAL-L253 and HAL-L132 (2.5–5%) (Fig. S1A).

In terms of the fungal communities, Ascomycota was the dominant phylum (81%), followed by Mucoromycota (13%), unassigned fungi (3%), Basidiomycota (2.8%), and Glomeromycota (0.75%) (Fig. S1B). The difference between RAS and RT was the enrichment of Mucoromycota in RAS (up to 38%) and Glomeromycota in RT (up to 4%). Inside the RT, a higher percentage of Basidiomycota (21%) was observed in HAL-L253 and Glomeromycota (4%) in HAL-L132, compared to the other PM lines (Fig. S1B). In alignment with the bacterial communities, fungal communities were different between PM lines only on the RT (Fig. S1B).

To identify differentially abundant taxa as biomarkers, we applied the linear discriminant analysis effect size (LEfSe) using the Kruskal–Wallis test (*p* < 0.05) with LDA score > 2.0. Notably, the RT of LAL-L220 and LAL-L3 lines showed specific colonization by certain Bacteroidetes species, while HAL lines exhibited specific colonization by species of Chloroflexi, *Paenibacillus*, and Actinobacteria. Additionally, the RAS of HAL lines exhibited several species belonging to Verrucomicrobia (Fig. 1C). In terms of the RT fungal community, of HAL lines displayed a distinct colonization pattern characterized by the presence of Basidiomycota species (Cantharellales). In contrast, the RT of LAL lines exhibited a unique colonization pattern with species from Mortiellomycota, including the genus Mortiella, and Mucuromycota, such as the genus *Rhizopus.* In the context of the RAS fungal community, both HAL and LAL lines displayed specific colonization patterns, each associated with a different genus of Ascomycota (Fig. 1D). These significant findings underscore the correlation between the composition of the rhizosphere microbial community and the soil’s aggregation capacity.

In terms of alpha-diversity, all compartments of PM lines (RAS and RT) exhibited a similar bacterial taxonomic richness compared to bulk soil (BS) across both methods (observed and Chao1, Fig. S2A–B) except for LAL-L220 in RT which showed a significantly higher level (*p* < 0.05). A similar trend was observed in the bacterial evenness between PM lines and BS but here in RAS the HAL-L253 showed a significantly (*p* < 0.05) reduced distribution calculated by both indices (Shannon and Simpson) and LAL-L220 in the Simpson index with respect to BS (Fig. S2C–D) evenness; PM lines of both compartments had similar levels to that of BS except for lines L220, L3, and L132 on RT which showed higher ones (Fig. S2G–H). Furthermore, we employed the Wilcoxon rank-sum test to construct rarefaction curves for the purpose of comparing bacterial and fungal diversity metrics within various compartments of each PM line. Both bacterial and fungal rarefaction curves displayed a clear plateau, signifying that the number of sequences analyzed was adequate to encompass the majority of OTUs in our study (Fig. SEIJ). There were no significant differences detected in bacterial alpha-diversity indices (richness and evenness) among the PM lines within compartments (Fig. S2A–D). In contrast, the fungal diversity in the RT compartment exhibited much higher evenness compared to BS and RAS, except for line HAL-L253 (Fig. S2E–H). This observation is corroborated by the data presented in the rarefaction curves (Fig. S2E, J).

### Microbial network analysis

**Fig. 2:**
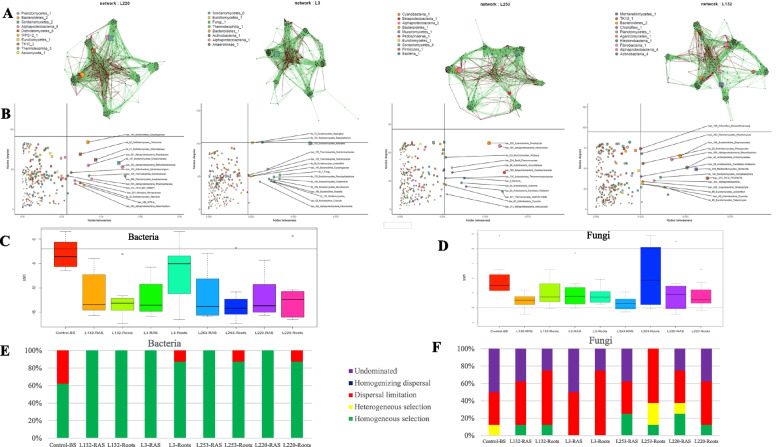
Microbial interaction network and assembly processes in pearl millet microbiome. **A** Co-occurrence network analysis illustrating the correlation of operational taxonomic units (OTUs) abundance within the bacterial and fungal communities in the roots (RT) and root-adhering soil (RAS) of four pearl millet PM lines (**A**, L220; **B**, L3, **C**, L253; **D**, L132). Each dot in the network represents a node, corresponding to a distinct OTU representing a microbial population. Strong Pearson correlations, filtered at a 0.05 *p*-value threshold, are represented by connections (green lines indicate a positive correlation while red lines indicate a negative correlation) between nodes. Nodes are color-coded and shaped according to their major taxonomic classes. The size of each node reflects its significance, determined by the number of connections (degree), betweenness, and closeness within the network. **B** Topological roles of classified nodes, revealing potential keystone species within the correlation network. **C**–**D** Assessment of relative contributions of deterministic (ßNTI ≥ 2) and stochastic (ßNTI ≤ 2) processes on the bacterial and fungal assembly across the soil–plant root continuum of the four PM lines, employing a null model. Horizontal lines indicate upper and lower significance thresholds at βNTI < 2 and > 2. **D** The relative importance of 5 ecological processes for bacterial and fungal communities assembly respectively, along the BS, RAS, and RT of each PM line: heterogeneous selection (ßNTI <  − 2), homogeneous selection (ßNTI > 2), dispersal limitation (|βNTI|< 2 and RC_Bray_ > heterogeneous selection (ßNTI <  − 2), homogeneous selection (ßNTI > 2), dispersal limitation (|βNTI|< 2 and RC_Bray_ > 0.95), homogenizing dispersal (|βNTI|< 2 and RC_Bray_ <  − 0.95), and undominated (|βNTI|< 2 and |RC_Bray_|< 0.95)

Microbiome changes were assessed through network analysis of each pearl millet (PM) line, utilizing 7000 to 10,000 edges (Fig. 2A). Microbial network analysis has been conducted using integrated data from both bacteria and fungi, and, to ensure accuracy, the abundances of RT and RAS compartments were merged from multiple data points, minimizing false positive and negative connections. The network structures were characterized by key indices including density, transitivity, diameter, average path length, number of modules, and edges (Table S1). Comparing these indices, the networks of LAL-L220 and LAL-L3 exhibited striking similarity, with < 2% variation observed across three out of the four indices. In contrast, the network of HAL-L253 differed significantly from the other PM lines, displaying the smallest network size, number of modules, mean connectivity, and average clustering, along with the highest average geodesic and modularity (Table S1).

The bacterial nodes within the networks of the four pearl millet (PM) lines were predominantly associated with Actinobacteria and Alphaproteobacteria (Fig. S3A), while the fungal nodes were primarily linked to Sordariomycetes and Dothideomycetes (Fig. S3B), with considerable overlap across the lines. However, the hubs, representing highly connected nodes, exhibited line-specific patterns within each compartment (Fig. 2B, S3C–D). The changing behavior of these hubs, as indicated by the number of positively and negatively shifting links, is visualized in a bar graph (Fig. S3E). Remarkably, the majority of nodes in all PM lines were peripheral or ultra-peripheral (specialists) based on their connectivity between and within modules in their network (Pi and Zi respectively, see material and method) (95.6% L220, 93.5% L3, 97.2% L253, and 95.2% L132) (Table S2). This implies that only a small portion of nodes function as connectors (generalists), with percentages ranging from 2.8 (HAL-L253) to 6.5% (LAL-L3) (Fig. 2B and Table S2).

### Microbial assembly processes

Ecological processes are fundamental in shaping microbial communities. The beta-nearest taxon index (ß-NTI) is a valuable tool for understanding the interplay between deterministic processes (e.g., environmental filtering, niche differentiation) and stochastic processes (e.g., dispersal limitation, ecological drift) in community turnover. Values of β-NTI greater than + 2 or less than − 2 indicate that community turnover is primarily driven by deterministic processes. On the other hand, β-NTI values between -2 and +2 are indicative of stochastic processes. By employing this approach, we were able to distinguish between the relative contributions of deterministic and stochastic processes in shaping microbial community dynamics. Indeed, an intriguing pattern emerges within the bacterial community in the RT and RAS compartments of the four PM lines, where ß-NTI values span a range from − 5 to − 15. This diversity implies a significant role of deterministic processes in shaping the assembly of bacterial communities (Fig. 2C). However, it is worth noting that bacterial taxa in the L3 RT exhibited a tendency toward stochastic assembly processes. In the bulk soil, there was also a subtle inclination toward a stochastic assembly process. The heightened involvement of homogeneous selection was evident in the assembly of bacterial communities within the RAS and RT compartments of each PM line. Conversely, the BS showed a more pronounced contribution of dispersal limitation, with the RT of L3, L220, and L253 demonstrating a milder impact of this factor (Fig. 2E). On the other hand, the fungal community exhibits a distinct trend for stochastic assembly processes, specifically dispersal limitation (Fig. 2D; F). Additionally, undominated processes played a significant role, except for the RT of L253 (Fig. 2F). Heterogeneous and homogeneous selection played variable roles, except in the case of the RAS and RT of L3 (Fig. 2F). These findings enhance our understanding of the factors driving microbial community dynamics in the rhizosphere and highlight the importance of deterministic processes in shaping plant-bacteria interactions.

### Metabonomics

To explore the relationship between soil aggregation capacity and metabolic profiles, we conducted in situ metabonomics analysis using FTICR-MS on 64 samples from different pearl millet (PM) lines. The analysis generated a data matrix comprising several thousand mass peaks, with approximately half assigned to molecules containing CHO-, CHNO-, or CHOS-elements within a molecular weight range of 137 to 701. Assignment criteria established by previous studies were employed [19–21], resulting in the assignment of 4217 molecular compositions. These compositions were visualized in van Krevelen plots and mass-edited H/C ratios (Fig. S4A–B), enabling their classification (Fig. S4C). CHO compositions accounted for 48% of the detected molecular compositions, with CHNO representing 47% and CHOS only 5% (Fig. S4A–B). Shoots exhibited a higher abundance of CHNO compositions, while the BS, RAS, and RT were enriched in CHO compositions and CHOS molecules, particularly in the RT (inserts in Fig. S4B).

We observed a slightly lower number of assigned formulae (1851) in the root-adhering soil (RAS) compared to the bulk soil (BS) control (1875 molecules) (Fig. S4A–B). This finding is consistent with studies conducted on *Arabidopsis thaliana* [22], and suggests that the microbial rhizosphere effect, influenced by root exudation, leads to a reduction in biochemical diversity in the rhizosphere [23–26]. The compounds detected in the BS and RAS compartments mainly comprised low-mass (150–400 m/z) CHO- and CHNO-molecules, identified as carbohydrates, aliphatics, and amino acids (Fig. S4B–C) [26]. In contrast, the compounds detected in the root tissues (RT) exhibited a lower number but a higher mass range (up to 700 m/z ratio) compared to the RAS and BS, with an increase in the CHOS molecular composition observed in the phenolic and carboxyl-rich aliphatic molecule (CRAM) zones, along with an increase in aliphatics (Fig. S4A–C). The shoot compartment of the PM lines showed the highest number of assigned molecules (2450) (Fig. S5B), which aligns with findings from studies on other plants such as rice and banana [27, 28]. Shoot samples exhibited a higher abundance of compounds in the aliphatic zone, particularly glycosylated carbohydrate-like and amino acids/peptide-like compounds, as well as highly unsaturated and condensed compounds associated with carboxyl-rich aliphatic molecules (CRAM) and phenolic classes (Fig. S4A–C).

In mass-edited H/C ratio plots, variations were observed in molecular compositions relatively with hydrogen-deficient covering the chemical space 0.5 ≤ H/C ≤ 1.0 and molecular masses 150 ≤ m/z ≤ 500 (Fig. S4B). In this region, modest changes (in %) in assigned chemical compositions between RAS (26%) and BS (29%) were noticed, while approximatively 17% and 20% were observed for shoots and roots, respectively. Overall, the variances of chemical fingerprints detected in shoots, roots, RAS, and BS demonstrated the specificity of each compartment. These differences in molecular composition between above- and below-ground plant parts can be attributed to factors such as biomass distribution, compartment-specific functions, cellular structures, and the production, translocation, and storage of primary and secondary metabolites [29–31].

**Fig. 3:**
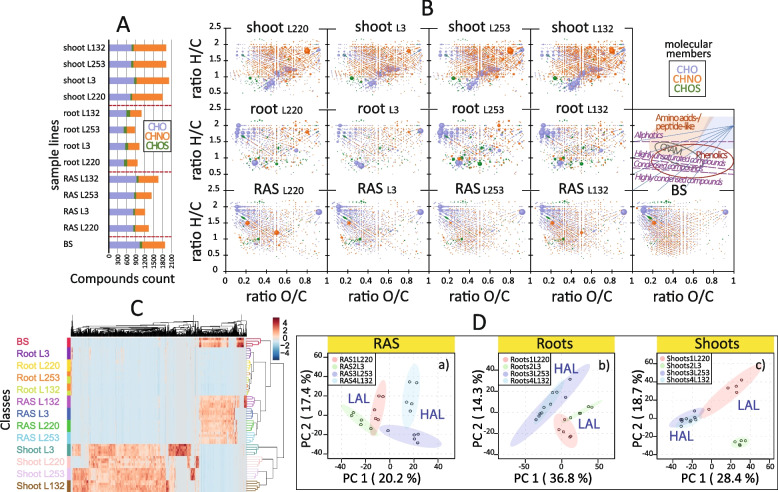
Comprehensive molecular composition analysis in pearl millet (PM) lines: unraveling variations across compartments. **A** Bar graph showing the distribution of CHO, CHNO, and CHOS compounds in the shoot, root, and root-adhering soil “RAS” compartments of the four PM lines (L220, L3, L253, and L132), as well as in the bulk soil “BS.” **B** H/C versus O/C van Krevelen diagrams depicting the distribution of all detected compounds from negative electrospray FTICR-MS analysis. The color codes represent different molecular compositions (blue CHO, orange CHNO, green CHOS), and the size of the bubbles indicates the signal intensity. The insert in the van Krevelen diagram highlights the classes of compounds. **C** Clustering heat map of the top 1000 significant compounds based on Pearson distance between spectra of the sixty-four samples (*p*-value ≤ 0.05). Each sample from each PM line in each compartment and the BS is indicated by a specific color in the margins. **D** PCA 2D scores plots illustrating the distribution of assigned compounds in different compartments of each PM line. Each sample is represented by a dot, and the color of the dots corresponds to a specific PM line. The 95% confidence region is displayed

Principal component analysis (PCA) revealed distinct differences between compartments of the pearl millet (PM) lines, as supported by the separation along the first two principal components PC1 (33%) and PC2 (15%). Specifically, shoots exhibited clear separation from other compartments in PC1, while RT and soil samples were distinct in PC2 (Fig. S4D). Additionally, PM lines with different soil aggregation capacities displayed distinct metabolomic profiles. The bulk soil (BS) compartment and shoots showed the highest diversity in compound composition, followed by the root-adhering soil (RAS), while RT exhibited the lowest diversity (Fig. 3A and B). Hierarchical clustering analysis based on significant features highlighted differences among the four PM lines (Fig. 3C). BS and RAS fractions exhibited similar total numbers of compounds (373 and 352, respectively) and composition (CHO > CHNO), while RT (248) displayed a balanced CHO/CHNO composition. Shoots displayed the highest number of assigned features (745), predominantly consisting of CHNO molecules (CHNO > CHO) (Fig. 3C). PCA analysis of the root exudate-containing fraction (RAS) demonstrated distinct clustering of PM lines along PC1 (20%) and PC2 (17%) (Fig. 3D). Specifically, in the root-adhering soil (RAS) compartment, the HAL lines (L132 and L253) exhibited the highest number of assigned carbohydrate (CHO) compounds compared to LAL lines (L3 and L220) (Fig. 3A). This indicates an increased presence of metabolites originating from root exudation and the RAS-associated microbiota, potentially contributing to enhanced soil aggregation in these HAL lines. Notably, there was a distinct differentiation between the two HAL lines, with HAL-L132 displaying a higher abundance of aliphatic CHO compounds compared to HAL-L253 (Fig. 3D). Previous studies on root exudation mechanisms have emphasized the inter- and intra-specific variability in root exudate composition as shown in *Helianthus* and *Quercus ilex* [32, 33].

In the RT compartment, the two HAL lines (L132 and L253) exhibited a distinct clustering pattern, appearing distant from LAL-L220 and LAL-L3 (Fig. 3D). Similarly, this trend was observed in the shoot compartment, where the two HAL lines clustered even further away from LAL-L3 compared to LAL-L220 (Fig. 3D). Root exudation mechanisms often involve a coupling of passive and active processes [24, 31, 34, 35].

The relationship between root exudate composition and soil aggregation in the rhizosphere was further supported by statistical analysis (PCA), which revealed clustering of samples and compartments of the four PM lines based on their aggregation capacity (Fig. 3D). Additionally, a heat map analysis of the 1000 most significant compounds (using Pearson distance), based on similarity values, demonstrated a clear correlation between the spectra of the 64 samples and the soil aggregation ratios of the PM lines in each compartment, particularly in the root and shoot compartments (Fig. 3C).

### Molecular specificities in PM lines

The BS compartment has the greatest number of unique low-mass CHNO- and CHO- molecules (200–500 m/z) compared to the RAS of the PM lines (Fig. S5C, Fig. S5A^RAS^-B^RAS^). These compounds correspond to the composition of low-mass range molecules found in the Bambey arenosol, which is characterized by low organic matter content (0.4% wt). To identify common root exudates and metabolites associated with microbial activities in the rhizosphere of each PM line, the composition of BS was subtracted from that of RAS. A total of 99 molecules ranging from 199 to 495 m/z mainly composed of carbohydrate/carbohydrate conjugates, amino acids/peptides and analogues, hydroxycinnamic acids and derivatives, fatty acids and conjugates, and fatty acid esters as well were found in all PM lines but absent in the BS.

Among a list of 37 molecules specific to HAL-L132 (Table S3), several were of interest, including coniferyl alcohol, known as a nod gene inducer in *Bradyrhizobium japonicum* [36], rhodojaponin, described as an insecticide produced by *Rhododendron molle* [37], hydroxyoxytetracycline, an antibiotic analog [38], cerulenin, an antifungal antibiotic isolated from *Cephalosporium caerulens* [39], and two homoserine lactones (N-(3-oxododecanoyl) homoserine lactone and N-(3-hydroxy-heptanoyl)-homoserine lactone), known to be involved in bacterial quorum-sensing [40].

Fewer specific molecules were identified in the other three PM lines: 10 for HAL-L253, 6 for LAL-L3, and 12 for LAL-L220. LAL-L220 harbored sieboldin, a dihydrochalcone compound typically found in *Malus* species [41], as well as lucidone A, a plant diterpene secondary metabolite. Fungal compounds predominantly produced by *Aspergillus* were retrieved from the RAS of the HAL line. Specifically, shoyuflavone A and a dihydropyranone called aspyrone were identified in the RAS of L253. Additionally, the RAS of L132 contained a sesquiterpenoid compound called asperugin and a carbonyl compound known as phomaligin. Notably, the RAS compartment of LAL-L3 and LAL-L220 contained two benzoxazinoids, DIMBOA-Glc (LAL-L3) and HDMBOA (LAL-L220) which have been found in maize [42] and more widely in *Poaceae* [43], knowing that pearl millet belongs to *Poaceae*.

### Correlation between metabarcoding and metabonomics datasets

**Fig. 4:**
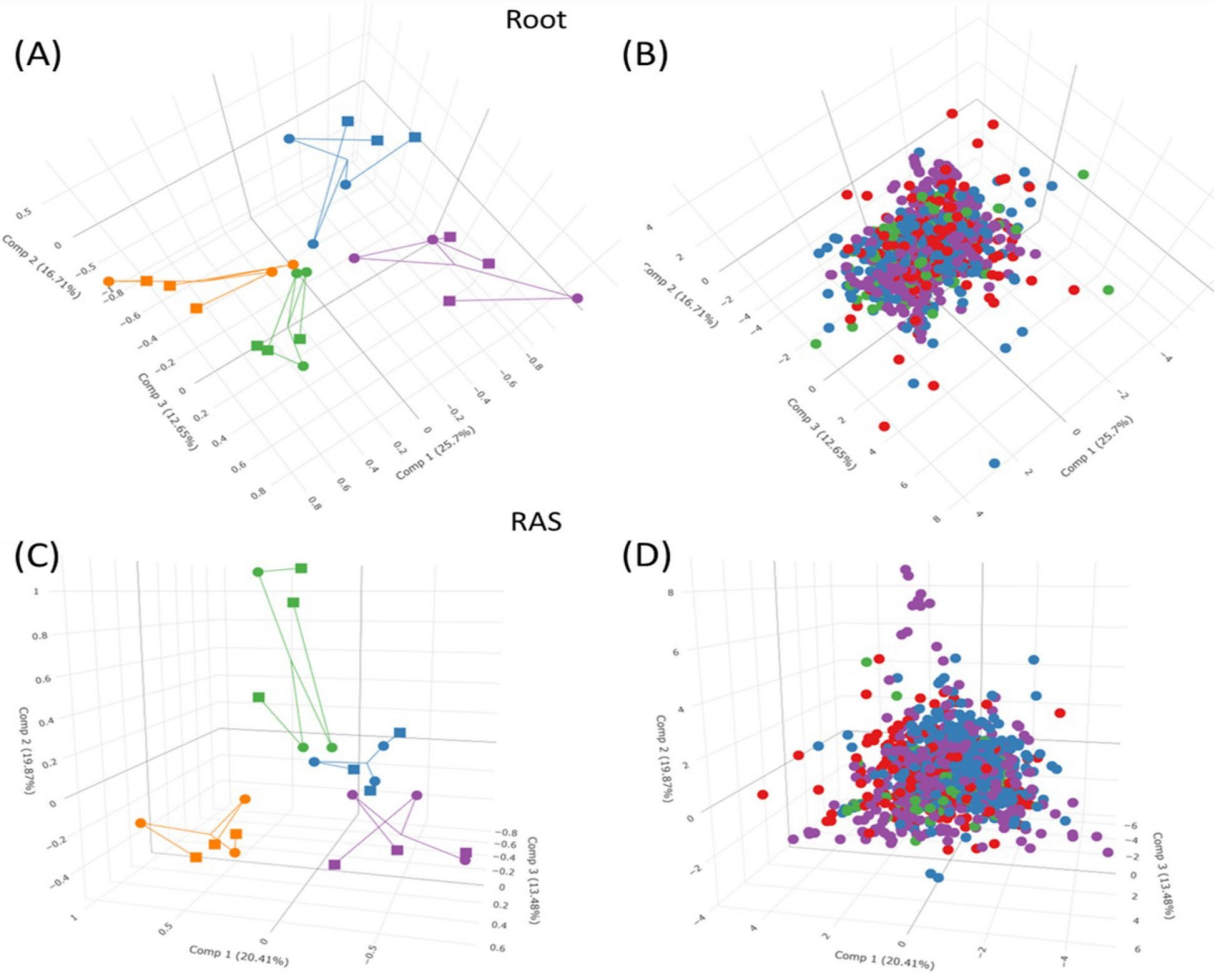
Deciphering microbiota-metabolite interactions in pearl millet “PM” lines: a co-inertia analysis (CIA) approach. CIA revealing the relationship between microbiota and metabolites in pearl millet “PM” lines. **A** and **C** 3D plots depicting the CIA of metabarcoding (represented by circular markers) and metabonomics (represented by square markers) data sets in the root and RAS compartments of the four PM lines (L200 in green, L3 in orange, L253 in purple, and L132 in blue). The lines connect the position of samples in the metabarcoding dataset with the corresponding position in the metabonomics dataset. **B** and **D** 3D plots illustrating the CIA of microbial OTUs (represented by purple markers) and the detected metabolites in the root and RAS compartments. The molecular formula of the metabolites is indicated by color-coding (CHO in red, CHNO in blue, and CHOS in green). CIA analysis, performed using R software

Co-inertia analysis of active microbial OTU abundance and the metabolite concentration covered in the RT and RAS compartments of each PM line revealed a correlation and covariation between these two datasets, influenced by the aggregation capacity (Fig. 4). The first two components explained approximately 40% of the total variance. In the RT compartment, components 1 and 2 accounted for 25.7% and 16.7% of the variance, while in the RAS compartment, they accounted for 20.4% and 19.8% of the variance. Notably, there was a clear separation between the HAL lines and the other two lines (IAL and LAL), which exhibited closer proximity to each other in the RT compartment than in the RAS (Fig. 4A–C). Co-inertia analysis was employed to evaluate the correlation between two matrices: one representing the abundance of OTUs, and the other depicting metabolite concentrations for each line. The resulting correlation coefficients (RV) were 0.672 and 0.635 for RT and RAS, respectively, both with a *p*-value of < 0.05, as determined through a Monte Carlo test involving 999 permutations. The visual representation in Fig. 4B and D vividly demonstrates that both OTUs and metabolites were co-positioned within the same spatial context as the PM line. This close spatial alignment emphasizes the strong connections between specific OTUs and metabolites within each line, implying a profound interrelationship between them.

## Discussion

Analysis of the alpha-diversity of the active microbiota revealed contrasting trends in bacterial and fungal communities between the RT and BS compartments, consistent with previous studies on plant diversity effects on soil microbiota [44–46].

Using the Bray–Curtis taxonomic dissimilarity index, we showed a clear separation between samples from BS, RAS, and RT compartments, in both bacterial and fungal communities, as reported in other plant studies [47]. Notably, PM line samples showed clustering and differentiation based on their aggregation capacity, particularly in the RT compartment, with distinct separation of HAL lines from LAL lines (Fig. 1A). The rhizosphere of HAL PM lines exhibited specific enrichment of Verrucomicrobia OTUs, suggesting their potential contribution to soil aggregation through increased root exudate availability [3].

The ß-NTI values elucidate that the assembly process of bacteria in the RT and RAS compartments of the four PM lines was markedly influenced by deterministic processes, predominantly characterized by a prevalence of homogeneous selection. This phenomenon, potentially driven by the presence of plant root exudates, underscores the pivotal role of these compounds in shaping bacterial community dynamics (Fig. 2C). An interesting parallel can be drawn from the research by Fan et al., revealing that the significance of deterministic processes in shaping diazotrophic communities diminishes as one moves away from wheat roots [48]. In a contrasting manner, the assembly of the fungal community within the pearl millet rhizosphere is primarily governed by stochastic processes (Fig. 2D). This outcome harmonizes with recent findings concerning the rhizosphere of *Typha orientalist* [49], as well as earlier studies [50, 51]. Depleted soils may promote the selection of microorganisms endowed with beneficial traits in relatively consistent environmental conditions, thereby diminishing the role of environmental filtering. This observation is consistent with a prior study [52, 53], which underscored the heightened significance of stochastic processes in shaping microbial community assembly during prolonged warming in a tall-grass prairie ecosystem, contrasting with deterministic processes [52]. Importantly, within unsaturated arenosol, fungi in such habitats may confront limited dispersal capacities, potentially leading to an increased degree of dispersal limitation.

The analysis of co-occurrence networks provides valuable insights into ecosystem functioning, plant nutrition, and resilience to biotic and abiotic stresses [54–56]. Our study revealed relatively minor differences in the organization and complexity of microbial networks among PM lines, suggesting that interactions are highly intricate in the rhizosphere (combined data from RT and RAS) of each line. The majority of specific connector hubs in each PM line network exhibited a combination of generalist and specialist characteristics, highlighting the role of the host plant in shaping the structure and function of its microbial community through root exudation [57, 58] (Fig. 2B). For instance, the *Mesorhizobium *genus(bac_283), known for EPS production [59], functioned as a generalist in the HAL network (Fig. S3 and 2B), potentially providing benefits to the plant, but shifted to a specialist role with different ecological implications in the LAL network (Fig. 2B).

Interactions among different species shape the assembly and functions of microbial communities, with potential beneficial, neutral, or detrimental effects on community members.

### Metabonomic profiles of PM line compartments

The influence of plant compartments on the microbiome, driven by their distinct physical and chemical properties, has been well documented [60, 61]. Previous studies have primarily focused on hydroponic or sterile plant systems, analyzing specific compounds related to root exudation, soil aggregation, and associated metabolites [62–66]. While these approaches successfully quantified specific chemicals of interest (e.g., phenolics, antioxidants) influencing plant growth and health [28, 67–69], they often overlooked the complexity of plant–microbe interactions and the biochemical diversity present in real-life conditions, where chemical compounds segregate among different plant parts [70].

In our study, we established an in situ system using native soil and employed a sensitive and untargeted analytical approach (FT-ICR-MS) to profile the diverse metabolites present in the various compartments of four PM lines with contrasting soil aggregation capacities. This allowed us to capture both root exudates and microbial metabolites activated by root exudation in the RAS compartment, as well as differentiate them from soil organic matter-related metabolites observed in the BS compartment. By considering the intricate interplay between plants and microbes under natural conditions, our approach provides a comprehensive understanding of the metabolic profiles associated with different plant compartments and their implications for plant–microbe interactions.

The RAS compartment of LAL-L3 and LAL-L220 contained two benzoxazinoids, DIMBOA-Glc (LAL-L3) and HDMBOA (LAL-L220). These secondary metabolites are known to trigger rhizosphere colonization by the plant-growth promoting bacterium *Pseudomonas putida* [71] and inhibit host recognition and virulence of the phytopathogen *Agrobacterium tumefaciens* [72]. More recently, using a metabonomic approach of root exudation in a non-sterile soil, it was shown that large amount of benzoxazinoids (including DIMBOA) and flavonoids were detected in the maize rhizosphere [73], and that benzoxazinoids (especially MBOA) shaped the bacterial and fungal diversity in the maize rhizosphere [74]. DIMBOA has also been shown to have multiple effects on rhizosphere microbiota, especially Proteobacteria and Chloroflexi*,* such as plant-soil feedback, metabolic regulation, and gatekeeper effects that will all lead to a change in the microbial community structure and functions [75, 76], and more recently, Wang et al. [77] showed that the exudations of GABA and DIMBOA are involved in shaping the rhizosphere and endosphere microbiomes. The absence of DIMBOA-Glc or HDMBOA-Glc in the RAS compartment of HAL lines may suggest that these molecules could negatively control the activity of soil-structuring bacteria in the rhizosphere of LAL lines (L3 and L220).

### Co-inertia analysis of RT and RAS omics datasets

Co-inertia analysis of the microbial populations and the specific metabolic compound datasets from the RT and RAS compartments revealed a correlation between the microbial populations and specific metabolic compounds in the PM lines, as depicted in the 3D plots (Fig. 4). The separation of the correlated omics data sets of each PM line and their alignment with the soil aggregation ratios further supported the link between plant-microbiota interactions and soil aggregation. Similar approaches combining metabonomics and metagenomics have been employed in various plant species, including *A. thaliana* [65, 74], *Avena barbata* [78], British bluebells [79], rice [80], tomato [66], potato [81], and poplar [82] highlighting the role of root exudation in shaping the root/rhizosphere-associated microbiota and their collective impact on soil aggregation. All of these studies mentioned above, as well as many others, have evidenced the effect of the plant through root exudation on the root/rhizosphere-associated microbiota and their combined role in soil aggregation [5, 83–89], which is under complex genetic control in pearl millet [18]. In the future, these omics approaches will continue to evolve and improve, particularly in terms of statistical and bioinformatics analysis [90–92], and combined with more complementary omics tools such as metaproteogenomics, metatranscriptomics and metaproteomics to strengthen the analysis of the plant-soil-microbiota continuum and shed light on this black box [82, 93–96]. This integration of diverse omics data will provide a more comprehensive understanding of the plant-soil-microbiota continuum, helping to unravel the complexities of this intricate relationship.

**Fig. 5:**
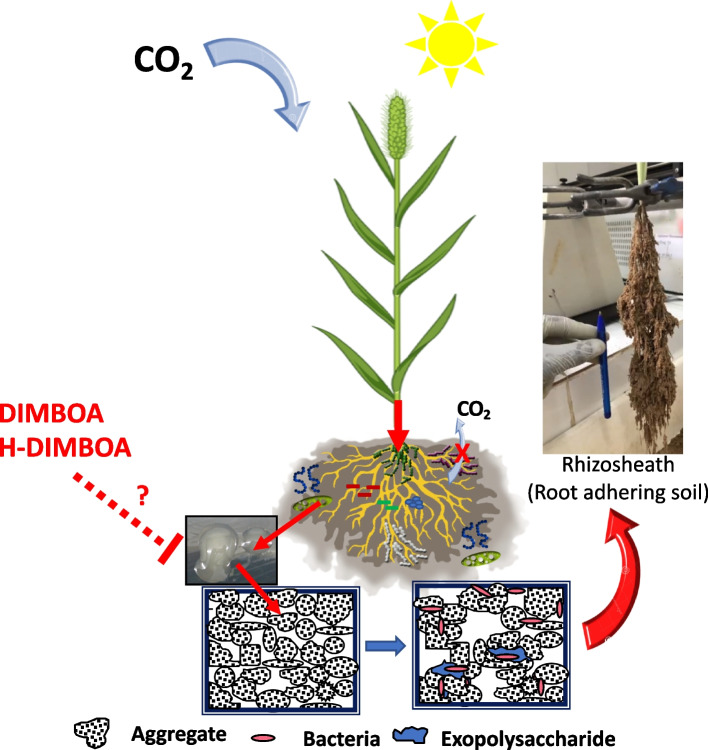
The ecological significance of root exudates: impact on rhizosphere microbiota and soil carbon dynamics root exudates play a critical role in shaping the assembly and interaction networks of the rhizosphere microbiota, which in turn influence the structure of the soil surrounding the roots. When certain microorganisms selected by the plant transform these exudates into exopolysaccharides (EPS), which act as cementing agents, enhancing soil adherence to the roots. Consequently, this process facilitates improved water and mineral supply to the plant and fosters carbon sequestration within the soil. DIMBOA and H-DIMBOA are exclusively present in the rhizosphere of LAL

An interplay of root features, encompassing factors such as root structure, development of root hairs, root architecture, root-associated microbiota, and partnerships with arbuscular mycorrhizal fungi, along with the secretion of root substances like exudates and mucilage, influences how soil adheres to and forms aggregates around the roots. The root exudate composition plays a crucial role in shaping the assembly and interaction networks of the rhizosphere microbiota, thereby influencing the structuring of the soil surrounding the roots (Fig. 5). A significant portion, up to 20%, of the photosynthetates produced by the plant is allocated as root exudates to recruit the rhizosphere microbiota. Some microorganisms transform these exudates into exopolysaccharides (EPS), which contribute to soil particle aggregation by increasing soil adherence to the roots. This process improves water and mineral availability for the plant and enhances carbon storage in the soil as the fresh carbon is not completely mineralized [3, 5]. Interestingly, the presence of DIMBOA and H-DIMBOA, known for their antimicrobial activity, is exclusively detected in the rhizosphere of PM lines with lower aggregation capacity. We hypothesize that these compounds may inhibit EPS synthesis by bacteria or selectively suppress certain EPS-producing bacterial populations. Further investigations are warranted to elucidate the mechanisms underlying the interplay between root exudate composition, EPS synthesis, and microbial communities, shedding light on their combined influence on soil aggregation and carbon sequestration in soils (Fig. 5). It is important to acknowledge that various other factors, including root architecture, root hairs, mucilage, priming-effect, and mycorrhizal fungi, are recognized as influencing rhizosheath formation.

## Conclusions

In summary, our investigation delved into the intricate relationship between root exudates, microbial communities, and soil aggregation in pearl millet (PM) lines. Analysis of alpha-diversity revealed contrasting trends in bacterial and fungal communities between root and bulk soil compartments, consistent with previous research on plant diversity effects on soil microbiota. Distinct assembly mechanisms governed the bacterial and fungal communities. Bacterial populations were chiefly shaped by deterministic processes, whereas the dynamics of fungal communities were primarily influenced by stochastic processes. The co-occurrence network analysis highlighted the role of root exudation in shaping the structure and function of the microbial community in the rhizosphere. Our in situ untargeted metabonomic approach provided comprehensive insights into the metabolic profiles associated with different plant compartments and their implications for plant–microbiota interactions. The correlation between microbial populations and specific metabolic compounds further emphasized the significance of root exudate composition in influencing soil aggregation. Overall, this study highlights the critical role of root exudates in modulating the assembly and function of rhizosphere microbiota, ultimately influencing soil aggregation and plant–microbiota interactions. Further research is needed to elucidate the mechanisms underlying these complex interactions to promote sustainable agriculture and effective strategies for soil carbon sequestration.

## Materials and methods

### Pearl millet line selection

Four pearl millet (*Pennisetum glaucum* (L.) R.Br.) (PM) inbred lines used in this study were selected based on their contrasted rhizosphere aggregation capacity as previously described [17]. Briefly, a screening experiment was performed on 181 PM lines by assessing their rhizosphere aggregation capacity, using the ratio of root-adhering soil (RAS) mass to root tissue (RT) mass (RAS/RT) to estimate rhizosheath [97]. PM lines L220, L3, L253, and L132 that differ in their RAS/RT ratio were chosen to study their metabonomics and its interaction with the RT and RAS-associated microbiota.

### Soil system description

The in situ soil-based system was set up in the Institut Sénégalais des Recherche Agricoles (ISRA) Bel Air campus in Dakar (Senegal), using native soil sampled in the Centre National de Recherche Agronomique (Bambey, Senegal). The soil was an arenosol (FAO classification) [97], which was sieved at 4 mm and homogenized before being distributed in 52 bottomless “WM” angular shaped pots that prevented root spiraling. The pots were divided into two sets of 28 pots (6 replicates for each of the 4 PM inbred lines and 4 as a control or bulk soil “BS”). Each pot contained 1.5 kg of soil. Watering was applied 3 times per week using 30 ml water for each pot for 28 days of growth. Then, the plants were carefully pulled out of the soil and fixed by their crown level on an electric agitator (Ingenieurbüro CAT M. Zipperer Shaker S 50 GmbH) and shaken at maximum speed for 1 min to detach the non-adhering soil. The roots were twice washed with 10 ml sterile distilled water to separate the RT from their RAS. For metabonomic analyses, four replicates of shoots, RT, RAS, and bulk soil (BS) were freeze-dried then processed by taking 50 mg of freeze-dried matter (fdm) from shoots, 10 mg fdm from RT, and 500 mg fdm from RAS. Prior to analysis, freeze-dried samples comprising 0.05 g of shoots, 0.01 g of RT, and 0.5 g of RAS were extracted using Precellys homogenizer (Bertin Technologies) with 1 ml of methanol (LC–MS grade, Fluka-Analytical,Sigma-Aldrich, St. Louis, USA) and deionized water (1:1 [vol/vol]) for 10 min. The samples were then centrifuged (25,000 g, 10 min, at room temperature) and the supernatant was collected and diluted in methanol (1:3 [vol/vol]). Analyses were performed at the Helmholtz- Munich, using a Fourier-Transform Ion Cyclotron Resonance Mass-Spectrophotometry Spectrometry (FT-ICR-MS).

For metabarcoding analyses, three replicates of RT, RAS, and BS were transferred to a sterile 15-ml tube containing 8 ml LifeGuard Soil Preservation solution (QIAGEN), then stored at − 80 °C until RNA extraction.

### FT-ICR-MS analyses

Ultrahigh-resolution mass spectra were acquired using FTICR-MS (solariX, Bruker Daltonics GmbH, Bremen, Germany) equipped with a 12-Tesla superconducting magnet (Magnex Scientific Inc., Yarnton, UK) and an APOLO II ESI source (Bruker Daltonics GmbH, Bremen, Germany) operating in negative ionization mode. Samples were introduced into the microelectrospray source at a flow rate of 120 μl h^−1^. Blanks (methanol) were run after every 8 samples to control cross-contamination and carry-over, and no interference during injections was observed. Spectra were acquired with a time domain of 4 MW over a mass range m/z of 92.1 to 1400, and 400 scans were accumulated per sample. Spectra were internally calibrated using the appropriate reference mass list, allowing mass accuracies of 0.1 ppm. Elemental compositions were assigned by a software tool written in-house [98]. Here, the assignments were generated based on the exact mass differences and the assigned molecular formulas were based on a restricted list of selected small molecular units with defined mass differences [98]. The compositional network enabled assignment of elemental formulas out of mass spectra and allowed alignments according to compositional relationships. The final elemental formulas were generated with a network tolerance of 0.2 ppm. The final elemental formulas were generated with a network tolerance of 0.2 ppm and classified into groups containing carbon, hydrogen, and oxygen atoms depending on the presence or absence of nitrogen and/or sulfur (molecular compositions CHO, CHNO, or CHOS), to reconstruct the group-selective mass spectra.

### RNA extraction and metabarcoding

Total RNA was extracted from 0.4 g of RT, and 2 g of RAS or BS using the RNeasy® PowerSoil® Total RNA Kit, Qiagen. DNA digestion was performed using the TURBO DNA-*free*™ Kit (Invitrogen) followed by RNA purification using the RNeasy® Mini Kit (Qiagen). cDNA was synthesized using the Transcriptor First Strand cDNA synthesis Ki, V.6 (Roche), and was used for PCR targeting bacteria and fungi, using the following primers: 515F-Y and 806RB for 16S rRNA gene, and fITS7 and ITS4, respectively. The primers were designed to contain overhang sequences compatible with Illumina Nextera XT index. The purified amplicons were sequenced by Biofidal Laboratory (Lyon, France) using the MiSeq, Illumina platform.

### Process of microbial community assembly

To explore the structure of bacterial community assembly processes by deterministic or stochastic processes, the β-nearest taxon index (βNTI; [99] was calculated using the R package “picante” (version 1.8.2). The β-NTI value, calculated using null-model expectations with consideration for phylogenetic distance, provides insight into the turnover of microbial communities. A null distribution of Beta Mean Nearest Taxon Distance (βMNTD) is performed by randomizing OTUs across the phylogeny and recalculating βMNTD 999 times [99]. βNTI quantifies the number of standard deviations that the observed βMNTD is from the mean of the null distribution. In our analysis, we quantified unweighted β-NTI values, without accounting for taxa relative abundances.

### Bioinformatics and statistics

The sequence data of 16S rRNA gene and ITS amplicons were analyzed using QIIME2 (qiime2:2019.10.0) [100]. The sequences were demultiplexed, denoised, and chimeras were removed using DADA2 [101]. The sequences were then aligned using MAFFT [102] and used to construct a phylogeny using FastTree [103]. Taxonomy was assigned using a naïve Bayes classifier trained on the GreenGenes 16S rRNA gene database (version 13_8) and the Unite ITS database (version 7). A total of 8213 features of 16S rRNA gene were generated from 212,986 reads from 28 samples (and with C1 sample filtered, the numbers were 7945,205,924 and 27 respectively). A total of 1412 features of ITS were generated from 780,825 reads from 28 samples (and with C1 sample filtered the numbers were 1355,735,565 and 27 respectively).

After generating the OTUs/sample abundance matrix, metadata file, and phylogenetic tree from Qiime 2 pipeline, they were uploaded to the MicrobiomeAnalyst server [104, 105]. Next, a data integrity check was performed using the “SanityCheckData” function, where OTUs with identical values (i.e., zero) in all samples and OTUs that appear in a single sample were excluded. Data filtering was then applied using the “ApplyAbundanceFilter” function based on the mean abundance value and the “*ApplyVarianceFilter*” function based on the standard deviation [106]. Data normalization was applied using the “*PerformNormalization*” function and data rarefied to the minimum library size [107]. We calculated alpha-diversity by taxonomic richness (observed OTUs) and the Chao1 index at a *p*-value less than 0.05. The Shannon and Simpson indices were used for estimation of evenness between samples at a *p*-value < 0.001. All diversity indices were compared among compartment lines using the *t*-test/ANOVA statistical method. For beta-diversity, a Bray–Curtis dissimilarity was used to measure the distance between each pair of samples. This explicit comparison of microbial communities (pairwise) based on their composition was tested using permutational multivariate analysis of variance (PERMANOVA,999 permutations) and plotted by the nonmetric multi-dimensional scaling (NMDS).

Linear discriminant analysis (LDA) coupled with the LDA effect size (LEfSe) technique was performed using LEfSe module for *huttenhower lab galaxy* [108]. The structure of abundance dataset was modified using R software to fit LEfSe module file format. The significance of Kruskal–Wallis and pairwise Wilcoxon tests were examined at the level of 0.05, and the threshold of LDA score was 2.

Microbial network analysis was performed by using the co-occurrence correlation of 16S rRNA gene and ITS abundance in RT and RAS for each PM line. Pearson correlation test was computed with R software to have the *p*-values and then corrected them by filtering with a *p*-value of 0.05 as threshold [109] resulting in a minimal correlation coefficient of 0.8. The resulting network topology and structure were computed using *igraph* package [110] on R software and described by a set of indices [111]. Extraction of hubs for each network by calculating the number of degree and the betweenness was then performed. The analysis of microbial network was completed by studying the topological roles of the nodes (including hubs) in the four PM lines by calculating among and within-module connectivity parameters Pi and Zi, respectively [112]. These parameters were used to determine peripheral (Zi < 2.5 and Pi < 0.62) and ultra-peripheral (Zi < 2.5 and Pi = 0) OTUs. Finally, in order to investigate the relation and interaction between metabarcoding and metabonomics datasets, a co-inertia analysis was performed with each metabonomic sample associated with its metabarcoding sample. Co-inertia was calculated with *mcoin* function from *omicade4* package [90]. The correlation coefficients (RV) were derived from the mcoin function, and a Montecarlo test was performed by repeatedly resampling metabarcoding and metabolomic matrices 999 times, as per the methodology outlined by [113]. Individual and variable coordinates were extracted, and their barycenters were calculated for each PM line. All coordinates were displayed in interactive 3D plots using the *plotly* package.

For metabonomics data analysis, statistically significant differences between groups of samples were evaluated using univariate ANOVA analyses (*p* < 0.05, FDR-corrected) to obtain significant m/z, performed in MetaboAnalyst [114]. Prior to this, aligned data were submitted to MetaboAnalyst, and for principal component analysis, peak intensities were normalized to the total ion count and scaled to unit-variance [115].

All statistical analyses were performed using MicrobiomeAnalyst (http://www.microbiomeanalyst.ca), MetaboAnalyst 4.0 (http://metaboanalyst.ca) and R v.3.6.2 (http://www.r-project.org/).

Raw data about 16S rRNA gene and ITS metabarcoding are stored in the FigShare platform (https://figshare.com/account/items/23635608/edit), and Raw data about metabonomic analyses are also stored in the FigShare platform (https://figshare.com/s/4dbf0ab5f7c6b2c9d41a).

### Supplementary Information

**Additional file 1:** **Figure S1.** Relative abundance of of OTUs of microbial communities (Phyla) in the different compartments (root and root adhering soil “RAS”) of the four pearl millet “PM” lines (L220, L3, L253, and L132) and bulk soil “BS”. A) Bar graph representing the relative abundance of bacterial community, B) Bar graph representing the relative abundance of fungal communities. Each color represents one of the major phyla. Each color refers to a condition (Compartment plus PM line). T-test ANOVA with p-value ≤ 0.05 for richness and < 0.001 for evenness. **Figure S2.** Alpha diversity of bacterial and fungal communities. A-H) Box plots of the microbial alpha-diversity in the different compartments (root and root adhering soil “RAS”) of the four-pearl millet “PM” lines (L220, L3, L253, and L132) and bulk soil “BS”; I-J) Rarefaction curves, Wilcoxon rank-sum test was used to compare the alpha-diversity index by using Shannon index to construct the rarefaction curves. **Figure S3.** Bacterial Network of the PM lines rhizosphere and roots microbiota. A) and B) Relative abundances of different bacterial and fungal  nodes and C) and D) Relative abundances of different bacterial and fungal hubs from both root and root adhering soil “RAS” compartments of pearl millet “PM” line networks (L220, L3, L253, and L132) and Venn diagrams indicating the number of nodes and hubs shared and not shared by the four PM lines in bacterial and fungal communities, respectively. E) Bar graphs representing the modifications of behavior of the microbial hubs in the four pearl millet “PM” lines (L220, L3, L253, and L132), the bacterial and fungal hubs shifting with the number of positive (green) and negative (red) links for each network of the PM lines. **Figure S4.** Representation of all molecular compositions of the assigned compounds as derived from negative electrospray FTICR-MS analysis in the bulk soil “BS” and in the different compartments (shoot, root, and root adhering soil “RAS”) of the four contrasting pearl millet “PM” lines samples in the *in situ* system. A) H/C versus O/C van Krevelen diagrams showing the distribution of the assigned compounds in the different compartments of the four PM lines and the BS (the color codes are blue, CHO; orange, CHNO and green, CHOS molecular compositions; bubble size is proportional to signal intensity). B) the corresponding mass-edited H/C ratios of the assigned compounds. Insert rings represent the relative propositions of the assigned CHO, CHOS, and CHNO molecular compositions including their computed numbers in each compartment and in BS.C) Van Krevelen diagram illustrating the classes of compounds. D) PCA 2D scores plots of the assigned compounds in all the PM lines samples in the different compartments and BS. The dots represent the samples where each compartment is symbolized by a color (red, BS; black, RAS; grey, shoot and green, root) with the display of 95% confidence region for each. **Figure S5.** Representation of discriminate molecular compositions specific for bulk soil “BS” and for each of the pearl millet “PM” line (L220, L3, L253, and L132) when compared to rest of the lines in each compartment (shoot, root and root adhering soil “RAS”). A) H/C versus O/C van Krevelen diagrams showing the distribution of the mass peaks of the unique molecular compositions in each line in each compartment and in the BS (the color codes are blue, CHO; orange, CHNO and green, CHOS). B) the corresponding mass-edited H/C ratios of the assigned unique compounds. C) Line graph representing the number of unique compounds (count of compounds) of CHO, CHNO and CHOS in each PM line in each compartment and in the BS. Yellow and blue rings highlight the differentiation between mass ranges of unique compounds*.*

**Additional file 2:** **Table S1**. Topological properties of major topological properties of the empirical phylogenetic Molecular Ecological Networks (pMENs) of microbial communities in the rhizosphere of the four pearl millet lines.

**Additional file 3:** **Table S2. **Table of microbial nodes distribution for each PM line (L220, L3, L253, and L132) showing the nodes numbers, proportions, and percentages over the zones (role) of the Within-module connectivity (Zi) over Among-module connectivity (Pi) score plot.

**Additional file 4.** Metabolites specific to L220 root exudates.

## Data Availability

Raw DNA sequence files and associated metadata were deposited in the US National Center for Biotechnology Information (NCBI) sequence read archive under accession number SAMN30841935. Raw data about 16S rRNA gene and ITS metabarcoding are stored in the FigShare platform (https://figshare.com/s/de6f384c2ae589a82d57), and raw data about metabonomic analyses are also stored in the FigShare platform (https://figshare.com/s/4dbf0ab5f7c6b2c9d41a). The remaining data that support the findings of this study are provided in Supplementary Data.
