# Supplementary material for: Unraveling the interplay between root exudates, microbiota, and rhizosheath formation in pearl millet
Source: Microbiome. 2024 Jan 3;12:1. doi: 10.1186/s40168-023-01727-3 (PMC10763007; doi:10.1186/s40168-023-01727-3)
Supplement: Supplementary file 2 — Additional file 1: Figure S1. Relative abundance of of OTUs of microbial communities (Phyla) in the different compartments (root and root adhering soil “RAS”) of the four pearl millet “PM” lines (L220, L3, L253, and L132) and bulk soil “BS”. A) Bar graph representing the relative abundance of bacterial community, B) Bar graph representing the relative abundance of fungal communities. Each color represents one of the major phyla. Each color refers to a condition (Compartment plus PM line). T-test ANOVA with p-value ≤ 0.05 for richness and < 0.001 for evenness. Figure S2. Alpha diversity of bacterial and fungal communities. A-H) Box plots of the microbial alpha-diversity in the different compartments (root and root adhering soil “RAS”) of the four-pearl millet “PM” lines (L220, L3, L253, and L132) and bulk soil “BS”; I-J) Rarefaction curves, Wilcoxon rank-sum test was used to compare the alpha-diversity index by using Shannon index to construct the rarefaction curves. Figure S3. Bacterial Network of the PM lines rhizosphere and roots microbiota. A) and B) Relative abundances of different bacterial and fungal nodes and C) and D) Relative abundances of different bacterial and fungal hubs from both root and root adhering soil “RAS” compartments of pearl millet “PM” line networks (L220, L3, L253, and L132) and Venn diagrams indicating the number of nodes and hubs shared and not shared by the four PM lines in bacterial and fungal communities, respectively. E) Bar graphs representing the modifications of behavior of the microbial hubs in the four pearl millet “PM” lines (L220, L3, L253, and L132), the bacterial and fungal hubs shifting with the number of positive (green) and negative (red) links for each network of the PM lines. Figure S4. Representation of all molecular compositions of the assigned compounds as derived from negative electrospray FTICR-MS analysis in the bulk soil “BS” and in the different compartments (shoot, root, and root adhering soil “RAS”) of the four [file 40168_2023_1727_MOESM1_ESM.pptx]

## Slide 1
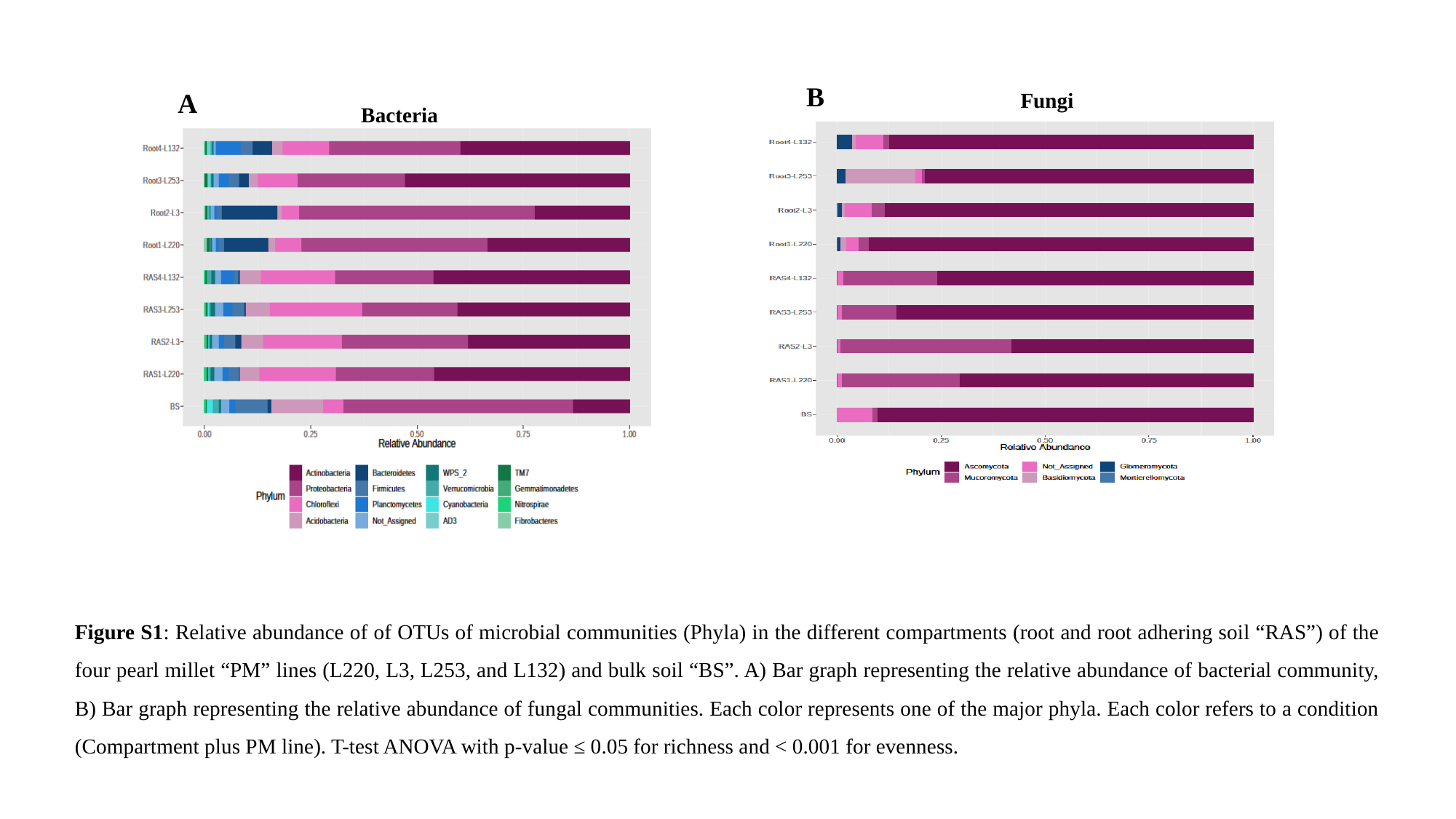

B
A
Fungi
Bacteria
Figure S1: Relative abundance of of OTUs of microbial communities (Phyla) in the different compartments (root and root adhering soil “RAS”) of the four pearl millet “PM” lines (L220, L3, L253, and L132) and bulk soil “BS”. A) Bar graph representing the relative abundance of bacterial community, B) Bar graph representing the relative abundance of fungal communities. Each color represents one of the major phyla. Each color refers to a condition (Compartment plus PM line). T-test ANOVA with p-value ≤ 0.05 for richness and < 0.001 for evenness.

## Slide 2
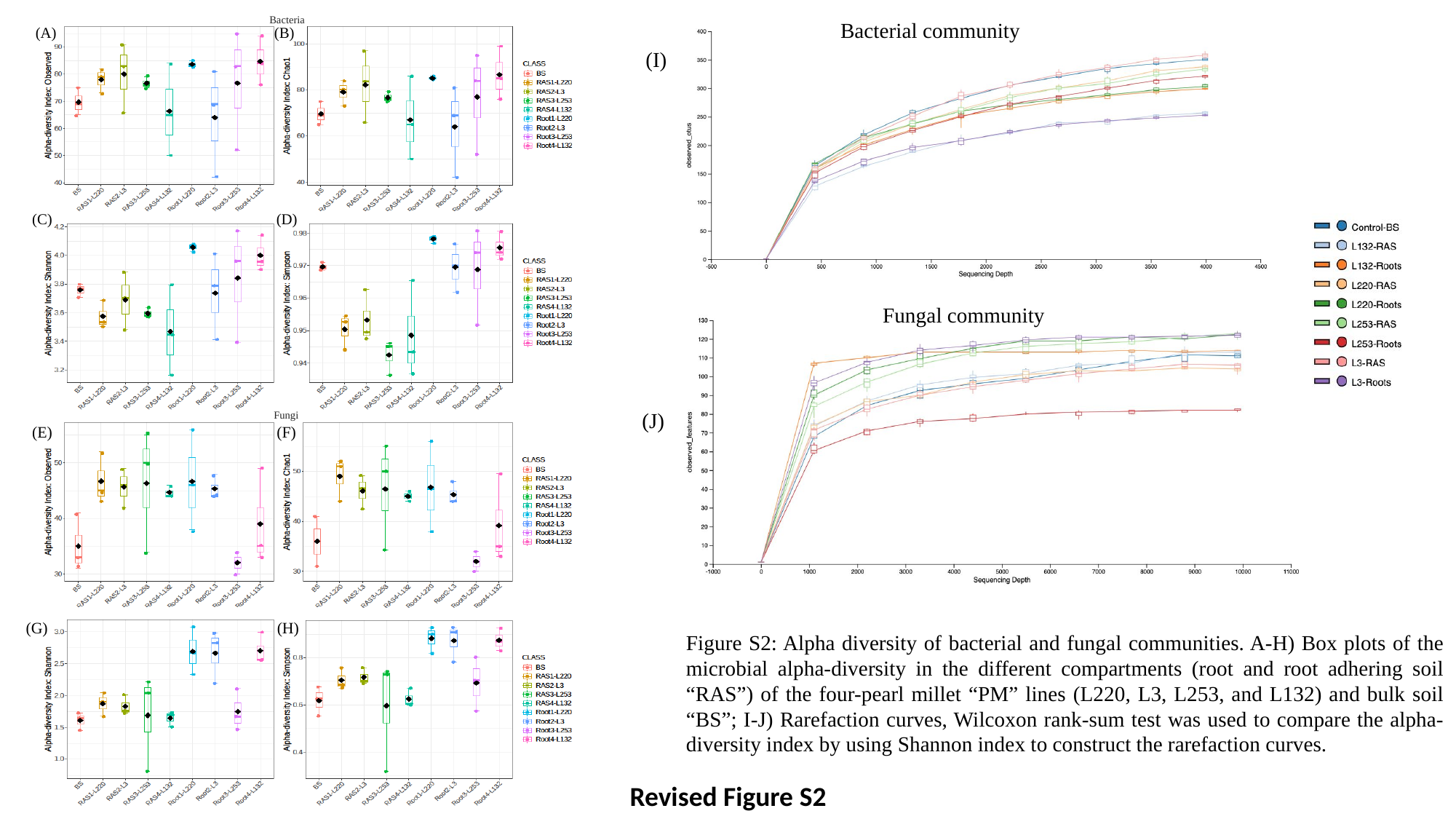

Bacteria
(A)
(B)
(D)
(C)
Fungi
(E)
(F)
(G)
(H)
Bacterial community
(I)
Fungal community
(J)
Figure S2: Alpha diversity of bacterial and fungal communities. A-H) Box plots of the microbial alpha-diversity in the different compartments (root and root adhering soil “RAS”) of the four-pearl millet “PM” lines (L220, L3, L253, and L132) and bulk soil “BS”; I-J) Rarefaction curves, Wilcoxon rank-sum test was used to compare the alpha-diversity index by using Shannon index to construct the rarefaction curves.
Revised Figure S2

## Slide 3
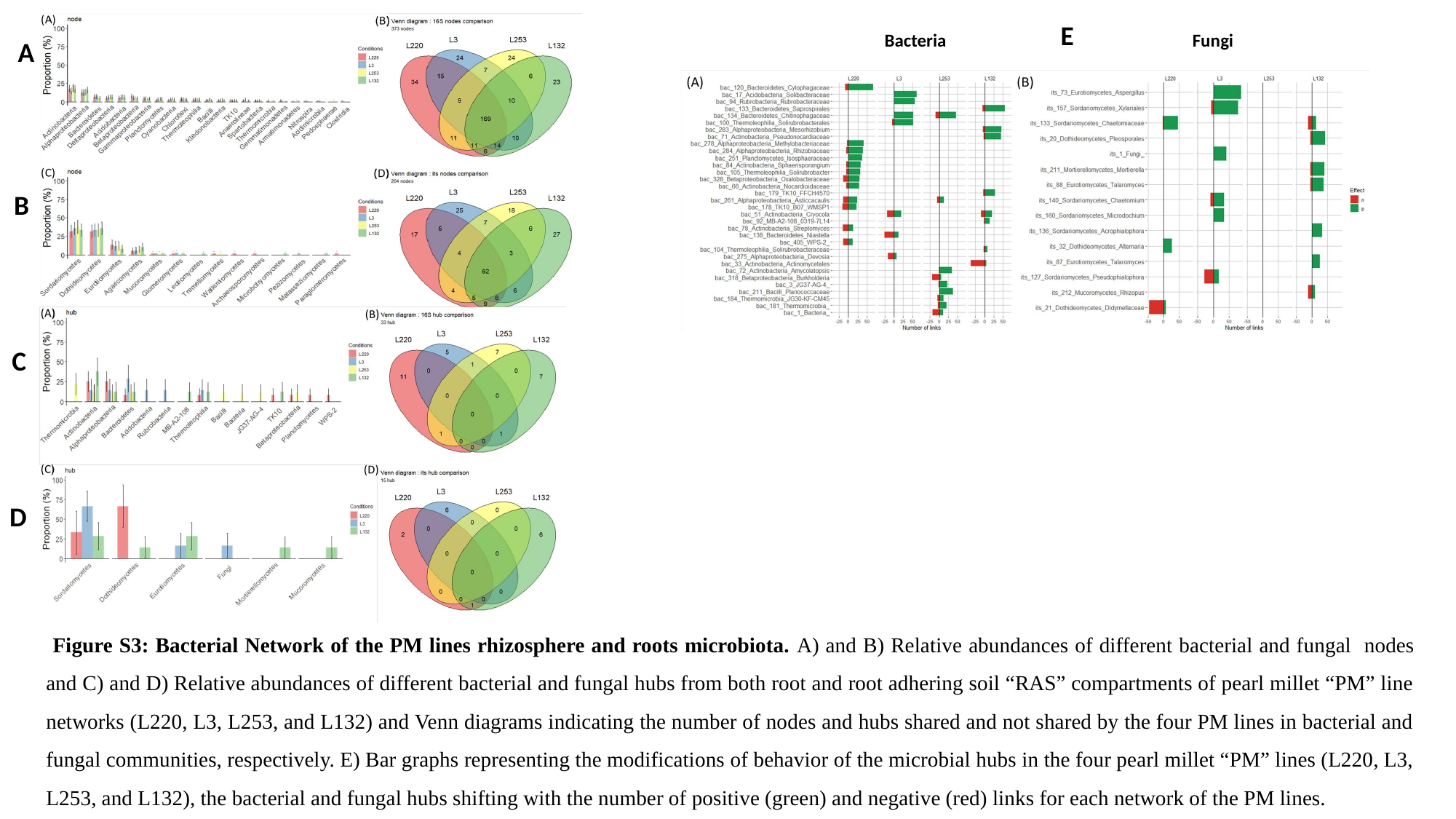

E
Bacteria
Fungi
A
B
C
D
 Figure S3: Bacterial Network of the PM lines rhizosphere and roots microbiota. A) and B) Relative abundances of different bacterial and fungal nodes and C) and D) Relative abundances of different bacterial and fungal hubs from both root and root adhering soil “RAS” compartments of pearl millet “PM” line networks (L220, L3, L253, and L132) and Venn diagrams indicating the number of nodes and hubs shared and not shared by the four PM lines in bacterial and fungal communities, respectively. E) Bar graphs representing the modifications of behavior of the microbial hubs in the four pearl millet “PM” lines (L220, L3, L253, and L132), the bacterial and fungal hubs shifting with the number of positive (green) and negative (red) links for each network of the PM lines.

## Slide 4
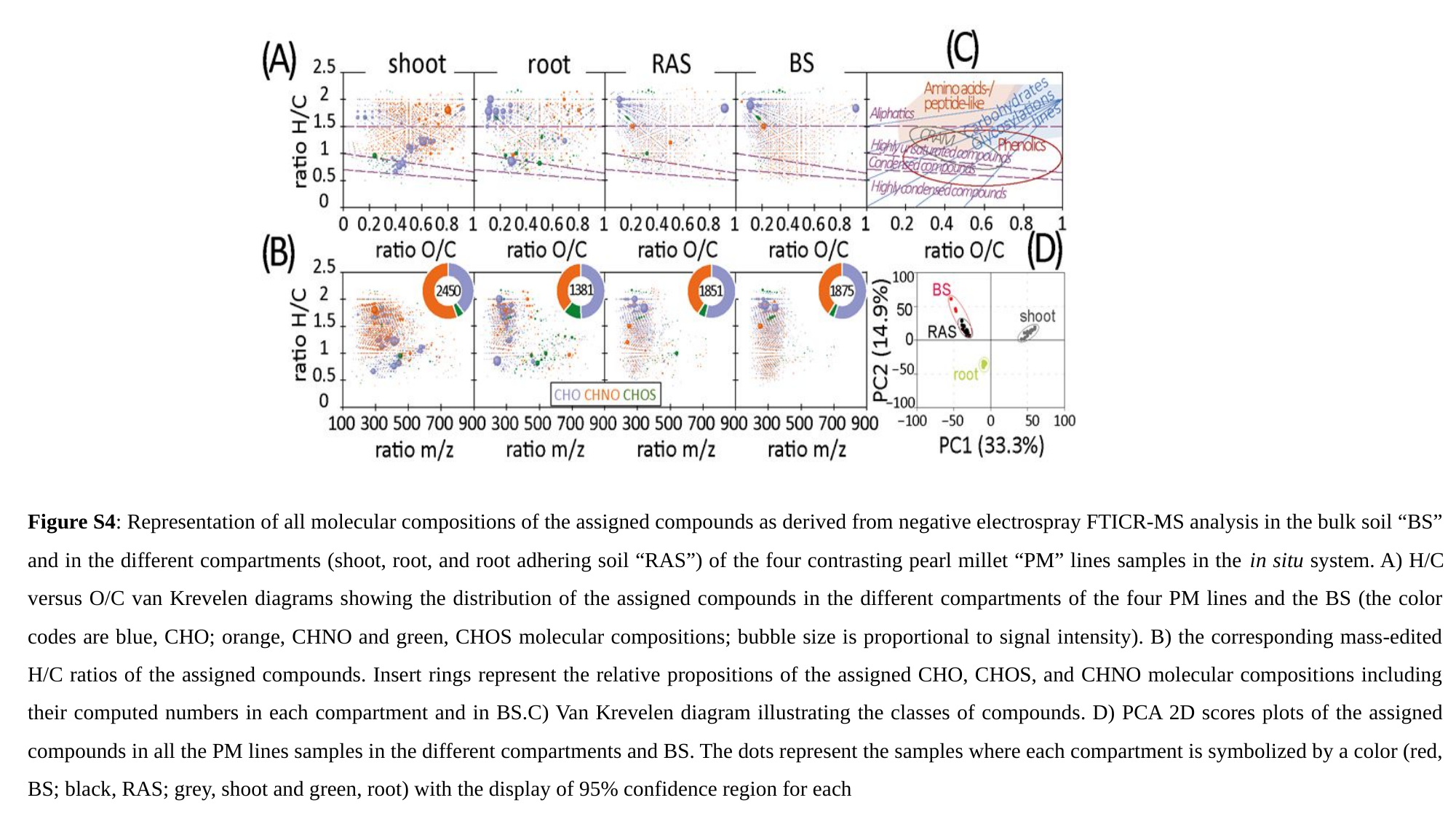

Figure S4: Representation of all molecular compositions of the assigned compounds as derived from negative electrospray FTICR-MS analysis in the bulk soil “BS” and in the different compartments (shoot, root, and root adhering soil “RAS”) of the four contrasting pearl millet “PM” lines samples in the in situ system. A) H/C versus O/C van Krevelen diagrams showing the distribution of the assigned compounds in the different compartments of the four PM lines and the BS (the color codes are blue, CHO; orange, CHNO and green, CHOS molecular compositions; bubble size is proportional to signal intensity). B) the corresponding mass-edited H/C ratios of the assigned compounds. Insert rings represent the relative propositions of the assigned CHO, CHOS, and CHNO molecular compositions including their computed numbers in each compartment and in BS.C) Van Krevelen diagram illustrating the classes of compounds. D) PCA 2D scores plots of the assigned compounds in all the PM lines samples in the different compartments and BS. The dots represent the samples where each compartment is symbolized by a color (red, BS; black, RAS; grey, shoot and green, root) with the display of 95% confidence region for each

## Slide 5
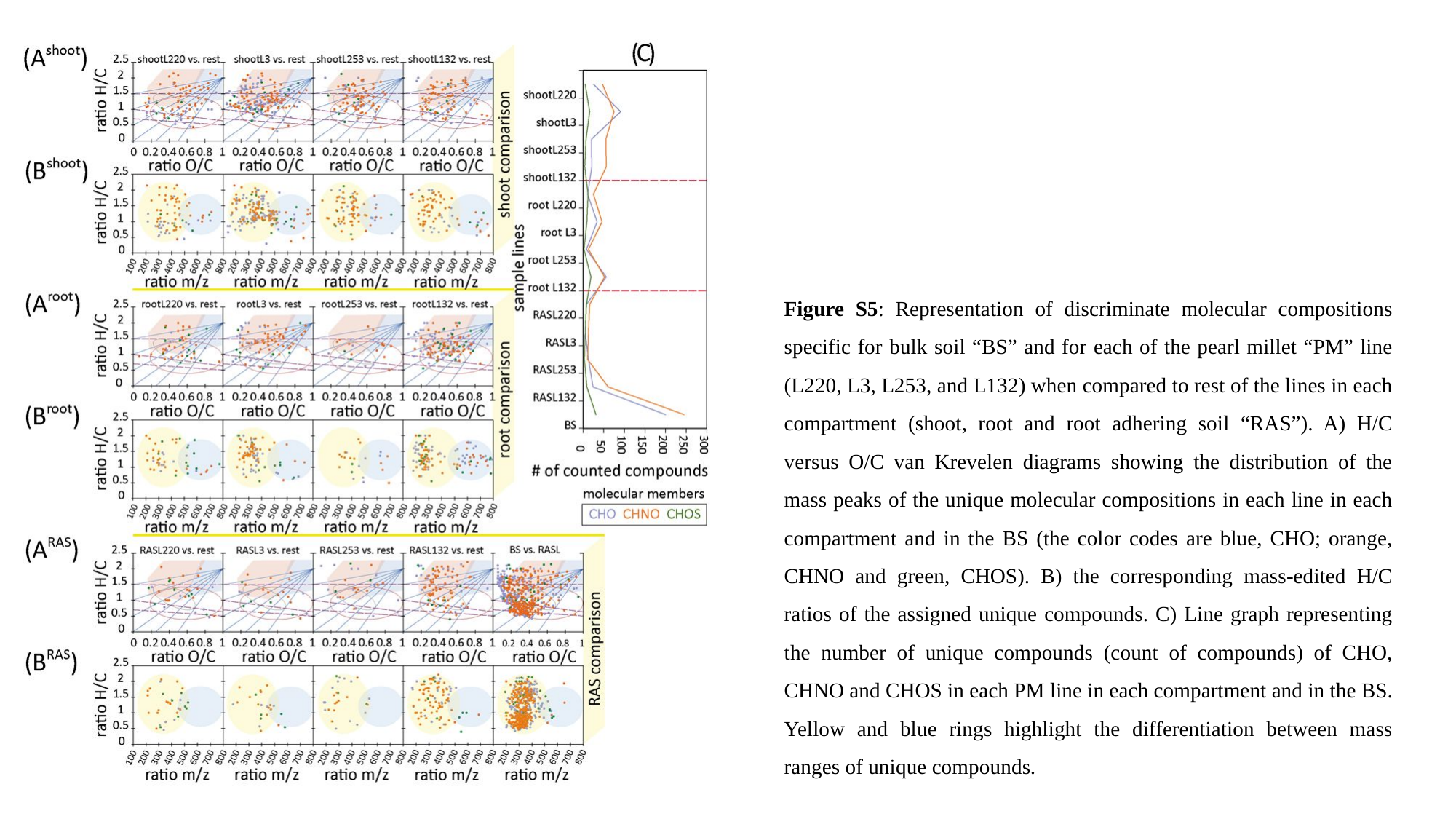

Figure S5: Representation of discriminate molecular compositions specific for bulk soil “BS” and for each of the pearl millet “PM” line (L220, L3, L253, and L132) when compared to rest of the lines in each compartment (shoot, root and root adhering soil “RAS”). A) H/C versus O/C van Krevelen diagrams showing the distribution of the mass peaks of the unique molecular compositions in each line in each compartment and in the BS (the color codes are blue, CHO; orange, CHNO and green, CHOS). B) the corresponding mass-edited H/C ratios of the assigned unique compounds. C) Line graph representing the number of unique compounds (count of compounds) of CHO, CHNO and CHOS in each PM line in each compartment and in the BS. Yellow and blue rings highlight the differentiation between mass ranges of unique compounds.
