# Supplementary material for: Unraveling the interplay between root exudates, microbiota, and rhizosheath formation in pearl millet
Source: Microbiome. 2024 Jan 3;12:1. doi: 10.1186/s40168-023-01727-3 (PMC10763007; doi:10.1186/s40168-023-01727-3)
Supplement: Supplementary file 3 — Additional file 2: Table S1. Topological properties of major topological properties of the empirical phylogenetic Molecular Ecological Networks (pMENs) of microbial communities in the rhizosphere of the four pearl millet lines. [file 40168_2023_1727_MOESM2_ESM.pdf]

Table S1: Topological properties of major topological properties of the empirical phylogenetic Molecular Ecological Networks (pMENs) of microbial communities in the rhizosphere of the four pearl millet lines.

	RAS-Root			
	**L220**	**L3**	**L132**	**L253**
Nb nodes	301	291	284	276
Nb edges	4344	4621	4288	3387
Density	0.10	0.11	0.11	0.09
Transitivity	0.77	0.84	0.86	0.84
Diameter	6	8	7	7
Average path length	3.08	3.12	3.26	3.30
