# Supplementary material for: Unraveling the interplay between root exudates, microbiota, and rhizosheath formation in pearl millet
Source: Microbiome. 2024 Jan 3;12:1. doi: 10.1186/s40168-023-01727-3 (PMC10763007; doi:10.1186/s40168-023-01727-3)
Supplement: Supplementary file 4 — Additional file 3: Table S2. Table of microbial nodes distribution for each PM line (L220, L3, L253, and L132) showing the nodes numbers, proportions, and percentages over the zones (role) of the Within-module connectivity (Zi) over Among-module connectivity (Pi) score plot. [file 40168_2023_1727_MOESM3_ESM.pdf]

Table S1: Table of microbial nodes distribution for each PM line (L220, L3, L253, and L132) showing the nodes numbers, proportions, and percentages over the zones (role) of the Within-module connectivity (Zi) over Among-module connectivity (Pi) score plot.

Plant line	Zone	Nodes per zone	Proportion	Percentage
L220	connector	16	0.04432133	4.43213296
	peripheral	158	0.43767313	43.767313
	ultra peripheral	187	0.51800554	51.800554
L3	connector	23	0.06497175	6.49717514
	peripheral	161	0.45480226	45.480226
	ultra peripheral	170	0.48022599	48.0225989
L253	connector	10	0.02808989	2.80898876
	peripheral	153	0.42977528	42.9775281
	ultra peripheral	193	0.54213483	54.2134832
L132	connector	18	0.04825737	4.82573727
	peripheral	143	0.38337802	38.3378016
	ultra peripheral	212	0.56836461	56.8364611
