# Supplementary material for: Unraveling the interplay between root exudates, microbiota, and rhizosheath formation in pearl millet
Source: Microbiome. 2024 Jan 3;12:1. doi: 10.1186/s40168-023-01727-3 (PMC10763007; doi:10.1186/s40168-023-01727-3)
Supplement: Supplementary file 5 — Additional file 4. Metabolites specific to L220 root exudates. [file 40168_2023_1727_MOESM4_ESM.pdf]

Metabolites specific to L220 root exudates

9.10^6

Specific molecules in RASL220 (N=12)								
Molecular.Weight	intensity.	Name (+N molecules with same MW)		Formula	Superclass	Class	Subclass	Parent.Level.1
404,2021	90052360	15beta-Hydroxy-7alpha-mercapto-pregn-4-ene-3,20-dione 7-acetate		C23H32O4!	Lipids and lipid-like molecules	Steroids and steroid derivative	Pregnane steroids	Gluco/mineralocorticoids, progestogins and de
402,2406	33169952	Lucidone A (+1)	Plants (Lauraceae)	C24H34O5	Lipids and lipid-like molecules	Steroids and steroid derivative	Oxosteroids	20-oxosteroids
452,1319	14037819	Sieboldin (+3)	Crassulaceae	C21H24O1	Phenylpropanoids and polyke	Flavonoids	Flavonoid glycosides	Flavonoid O-glycosides
516,0938	11196639	[4,5-dihydroxy-6-(hydroxymethyl)-2-{2,4,6-trihydroxy-3-[3-(3-hydroxyphenyl)propanoyl]phenyl}oxan-3-yl]oxidanesulfonic acid (+2)		C21H24O1	Phenylpropanoids and polyke	Linear 1,3-diarylpropanoids	Chalcones and dihydrochalcones	2'-Hydroxy-dihydrochalcones
528,1665	5475266	17-beta-estradiol 3-sulfate-17-(beta-D-glucuronide)		C24H32O1	Lipids and lipid-like molecules	Steroids and steroid derivative	Steroidal glycosides	Steroidal glycosides
422,1002	4841861	Thonningine A (+1)	Seeds of Millettia thonningii	C23H18O8	Phenylpropanoids and polyke	Coumarins and derivatives	Furanocoumarins	Linear furanocoumarins
442,1376	4000705	Methacycline	Tetracyclin	C22H22N2	Phenylpropanoids and polyke	Tetracyclines	NA	Tetracyclines
389,1838	3623412	3-(3,4-dimethoxyphenyl)-N-[2-(3,4-dimethoxyphenyl)ethyl]-2-hydroxypropanimidic acid		C21H27NO	Benzenoids	Benzene and substituted deriv	Methoxybenzenes	Dimethoxybenzenes
222,0641	3066538	Metoxadiazone	Insecticide	C10H10N2	Benzenoids	Benzene and substituted deriv	Aniline and substituted anilines	Methoxyanilines
215,1158	2879533	6-hydroxyoct-2-enoylglycine		C10H17NO	Organic acids and derivatives	Carboxylic acids and derivative	Amino acids, peptides, and analogues	Amino acids and derivatives
387,1165	2822383	**HDMBOA-Glc**	Powerful antibiotic present in maize, wheat, and related grasses	C16H21NO	Organic oxygen compounds	Organooxygen compounds	Carbohydrates and carbohydrate con	Glycosyl compounds
232,1059	2513698	N2-Succinyl-L-ornithine (+1)		C9H16N2O	Organic acids and derivatives	Carboxylic acids and derivative	Amino acids, peptides, and analogues	Amino acids and derivatives

Metabolites specific to L3 root exudates

Specific molecules in RASL3 (N=6)								
Molecular.Weight	intensity.	Name (+N molecules with same MW)		Formula	Superclass	Class	Subclass	Parent.Level.1
398,2093	4145704	Asacoumarin A (+3)	Root or rhizome of Ferula assa-foetida (Apiacées, plant)	C24H30O5	Lipids and lipid-like molecules	Prenol lipids	Terpene lactones	Terpene lactones
308,1736	3264912	Ubenimex	Microbial metabolite and dipeptide	C16H24N2(	Organic acids and derivatives	Peptidomimetics	Hybrid peptides	Hybrid peptides
373,1009	3036472	**DIMBOA-Glc**	Powerful antibiotic present in maize, wheat, and related grasses	C15H19NO	Organic oxygen compounds	Organooxygen compounds	Carbohydrates and carbohydrate con	Glycosyl compounds
442,1992	2873064	Exiguaflavanone M	Root of Sophora exigua	C25H30O7	Phenylpropanoids and polyket	Flavonoids	Flavans	8-prenylated flavans
228,1474	2270805	Isoleucylproline		C11H20N2(	Organic acids and derivatives	Carboxylic acids and derivative	Amino acids, peptides, and analogues	Peptides
178,0994	2216714	Methylisoeugenol (+11)	Plant roots	C11H14O2	Benzenoids	Benzene and substituted deriv	Methoxybenzenes	Dimethoxybenzenes

Metabolites specific to L253 root exudates

Specific molecules in RASL253 (N=10)								
Molecular.Weight	intensity.	Name (+N molecules with same MW)		Formula	Superclass	Class	Subclass	Parent.Level.1
262,0801	3692688	Glutamylaspartic acid		C9H14N2O	Organic acids and derivatives	Peptidomimetics	Hybrid peptides	Hybrid peptides
304,2614	3233255	1-O-(2R-hydroxy-tetradecyl)-sn-glycerol		C17H36O4	Lipids and lipid-like molecules	Fatty Acyls	Fatty alcohols	Long-chain fatty alcohols
386,0638	2836969	Shoyuflavone A	Aspergillus	C19H14O9	Phenylpropanoids and polyke	Isoflavonoids	Isoflav-2-enes	Isoflavones
342,3498	2726800	1,2-docasanediol		C22H46O2	Lipids and lipid-like molecules	Fatty Acyls	Fatty alcohols	Fatty alcohols
292,1886	2703215	(S)-3-Octanol glucoside		C14H28O6	Lipids and lipid-like molecules	Fatty Acyls	Fatty acyl glycosides	Fatty acyl glycosides of mono- and disaccharid
250,1053	2392221	Isopropyl citrate		C10H18O7	Organic acids and derivatives	Keto acids and derivatives	Medium-chain keto acids and derivat	Medium-chain keto acids and derivatives
248,0433	2355663	(E)-2-(2-Furyl)-3-(5-nitro-2-furyl)acrylamide		C11H8N2O	Organoheterocyclic compour	Furans	Nitrofurans	Nitrofurans
192,1150	2033557	Thymyl acetate (+9)	Plants	C12H16O2	Lipids and lipid-like molecules	Prenol lipids	Monoterpenoids	Aromatic monoterpenoids
184,0736	1910625	Aspyrone (+4)	Aspergillus	C9H12O4	Organoheterocyclic compour	Pyrans	Pyranones and derivatives	Dihydropyranones
192,0786	1683526	Allyl phenoxyacetate (+8)		C11H12O3	Benzenoids	Benzene and substituted deriv	Phenoxyacetic acid derivatives	Phenoxyacetic acid derivatives

Metabolites specific to L132 root exudates

Specific molecules in RASL132 (N=37)

Molecular.Weight	intensity.	Name (+N molecules with same MW)		Formula	Superclass	Class	Subclass	Parent.Level.1
402,1162	10941561	6-[5-(4-carboxy-2-hydroxybutyl)-2-hydroxyphenoxy]-3,4,5-trihydroxyoxane-2-carboxylic acid		C17H22O11	Lipids and lipid-like molecules	Saccharolipids	NA	Saccharolipids
400,2250	9001824	Asperugin	Aspergillus	C24H32O5	Lipids and lipid-like molecules	Prenol lipids	Sesquiterpenoids	Sesquiterpenoids
373,1525	8495940	1,2-O-Diacetylzephyranthine	Bulbs of Cyrtanthus elatus	C20H23NO6	Alkaloids and derivatives	Amaryllidaceae alkaloids	Lycorine-type amaryllidaceae alkaloids	Lycorine-type amaryllidaceae alkaloids
393,1212	7365178	Dihydromacarpine;<br>13,14-Dihydro-5,7-dimethoxy-13-methyl-[1,3]benzodioxolo[5,6-c]-1,3-dioxolo[4,5-i]phenanthridine		C22H19NO6	Alkaloids and derivatives	Benzophenanthridine alkaloids	Dihydrobenzophenanthridine alkaloids	Dihydrobenzophenanthridine alkaloids
454,2567	5321153	Rhodojaponin IV	Botanical insecticide	C24H38O8	Lipids and lipid-like molecules	Prenol lipids	Diterpenoids	Grayanoids
268,0695	4799820	Portulacaxanthin III	Natural betaxanthins that occur in the flowers of Portulaca grandiflora.	C11H12N2O6	Organic acids and derivatives	Carboxylic acids and derivatives	Amino acids, peptides, and analogues	Amino acids and derivatives
476,1431	4447301	11a-Hydroxyoxytetracycline	Antobiotic?	C22H24N2O1	NA	NA	NA	NA
292,0617	4379506	S-methylazathioprine		C10H10N7O2	Organoheterocyclic compounds	Imidazopyrimidines	Purines and purine derivatives	Purines and purine derivatives
346,1264	4368417	Aucubin (+1)	Ginko et plantain	C15H22O9	Lipids and lipid-like molecules	Prenol lipids	Terpene glycosides	Iridoid O-glycosides
380,2410	3747106	Linusic acid		C18H36O8	Lipids and lipid-like molecules	Fatty Acyls	Fatty acids and conjugates	Long-chain fatty acids
388,3553	3650236	1-O-(2R-hydroxy-eicosanyl)-sn-glycerol		C23H48O4	Lipids and lipid-like molecules	Fatty Acyls	Fatty alcohols	Long-chain fatty alcohols
311,1733	3622471	Phomaligin A	Aspergillus	C16H25NO5	Organic oxygen compounds	Organooxygen compounds	Carbonyl compounds	Acyloins
361,1162	3545402	Avenanthramide A2	Avoine	C18H19NO7	Phenylpropanoids and polyketides	Cinnamic acids and derivatives	Hydroxycinnamic acids and derivatives	Hydroxycinnamic acids and derivatives
427,1478	3505951	(S)-2-Hydroxy-2-phenylacetonitrile O-[b-D-apiosyl-(1->2)-b-D-glucoside]		C19H25NO1(	Organic oxygen compounds	Organooxygen compounds	Carbohydrates and carbohydrate conjugate	Glycosyl compounds
387,1682	3326981	N-[2-(3,4-dimethoxyphenyl)ethyl]-3-(2-hydroxy-3,4-dimethoxyphenyl)prop-2-enimidic acid		C21H25NO6	Benzenoids	Phenols	Methoxyphenols	Methoxyphenols
245,1376	3296721	Asparaginyl-Leucine		C10H19N3O4	Organic acids and derivatives	Carboxylic acids and derivatives	Amino acids, peptides, and analogues	Peptides
297,1940	3274065	N-(3-oxododecanoyl) homoserine lactone	Bacteria Quorum-sensing	C16H27NO4	Organic acids and derivatives	Carboxylic acids and derivatives	Amino acids, peptides, and analogues	Amino acids and derivatives
436,3553	3180321	5alpha-Cholestane-3alpha,7alpha,12alpha,24-tetrol		C27H48O4	Lipids and lipid-like molecules	Steroids and steroid derivatives	Bile acids, alcohols and derivatives	Hydroxy bile acids, alcohols and derivatives
346,3083	3133495	1-O-(2R-methoxy-hexadecyl)-sn-glycerol		C20H42O4	Lipids and lipid-like molecules	Glycerolipids	Monoradylglycerols	Monoalkylglycerols
365,1688	3064967	Trimethaphan	Vasodilator agent	C22H25N2OS	Organoheterocyclic compounds	Thienoimidazolidines	NA	Thienoimidazolidines
264,1573	2925825	Hexyl glucoside		C12H24O6	Lipids and lipid-like molecules	Fatty Acyls	Fatty acyl glycosides	Fatty acyl glycosides of mono- and disaccharides
411,2257	2913005	Lasiocarpine<br>7-Angelyl-9-lasiocarpylheliotridine		C21H33NO7	Alkaloids and derivatives	NA	NA	Alkaloids and derivatives
174,0892	2880093	Diethyl succinate		C8H14O4	Lipids and lipid-like molecules	Fatty Acyls	Fatty acid esters	Fatty acid esters
308,1372	2835111	2-Carboxy-1-[5-(2-carboxy-1-pyrrolidinyl)-2-hydroxy-2,4-pentadienylidene]pyrrolidinium		C15H20N2O5	Organic acids and derivatives	Carboxylic acids and derivatives	Amino acids, peptides, and analogues	Amino acids and derivatives
394,0536	2679720	3,4,5-trihydroxy-6-({8-hydroxy-2-oxo-2H-furo[2,3-h]chromen-4-yl}oxy)oxane-2-carboxylic acid		C17H14O11	Phenylpropanoids and polyketides	Coumarins and derivatives	Coumarin glycosides	Coumarin glycosides
322,0325	2651224	3,4-dihydroxy-5-(3,4,5-trihydroxybenzoyloxy)benzoic acid		C14H10O9	Phenylpropanoids and polyketides	Depsides and depsidones	NA	Depsides and depsidones
170,1307	2608280	9-Hydroxygeraniol (+5)	Citrus, Eucalyptus, Juniperus and Pinus	C10H18O2	Lipids and lipid-like molecules	Prenol lipids	Monoterpenoids	Acyclic monoterpenoids
195,0532	2503512	Dopaquinone (+1)	Salicornia	C9H9NO4	Organic acids and derivatives	Carboxylic acids and derivatives	Amino acids, peptides, and analogues	Amino acids and derivatives
333,1325	2338356	gamma-Glutamyltryptophan		C16H19N3O5	Organic acids and derivatives	Carboxylic acids and derivatives	Amino acids, peptides, and analogues	Peptides
260,1008	2334661	L-alpha-glutamyl-L-hydroxyproline		C10H16N2O6	Organic acids and derivatives	Carboxylic acids and derivatives	Amino acids, peptides, and analogues	Peptides
255,1471	2235700	Meteloidine	Brugmansia and Datura species	C13H21NO4	Alkaloids and derivatives	Tropane alkaloids	NA	Tropane alkaloids
261,1212	2161968	Lotaustralin	Lotus Manihot Phaseolus Trifolium	C11H19NO6	Organic oxygen compounds	Organooxygen compounds	Carbohydrates and carbohydrate conjugate	Glycosyl compounds
229,1314	2137772	N-(3-hydroxy-heptanoyl)-homoserine lactone	Bacteria Quorum-sensing	C11H19NO4	Organic acids and derivatives	Carboxylic acids and derivatives	Amino acids, peptides, and analogues	Amino acids and derivatives
181,0739	2107827	Beta-Tyrosine		C9H11NO3	Organic acids and derivatives	Carboxylic acids and derivatives	Amino acids, peptides, and analogues	Amino acids and derivatives
180,0786	1981249	Coniferyl alcohol (+2)	Plants	C10H12O3	Benzenoids	Phenols	Methoxyphenols	Methoxyphenols
219,0532	1904538	5-Hydroxyindolepyruvate (+2)		C11H9NO4	Organoheterocyclic compounds	Indoles and derivatives	Indolyl carboxylic acids and derivatives	Indolyl carboxylic acids and derivatives
223,1208	1798764	Cerulenin (+2)	Antifungal antibiotic from Cephalosporium caerulens (Ascomycetes)	C12H17NO3	Organoheterocyclic compounds	Epoxides	Oxirane carboxylic acids and derivatives	Oxirane carboxylic acids and derivatives
